# A Functional Polymorphism in the *5HTR2C* Gene Associated with Stress Responses Also Predicts Incident Cardiovascular Events

**DOI:** 10.1371/journal.pone.0082781

**Published:** 2013-12-18

**Authors:** Beverly H. Brummett, Michael A. Babyak, Rong Jiang, Svati H. Shah, Richard C. Becker, Carol Haynes, Megan Chryst-Ladd, Damian M. Craig, Elizabeth R. Hauser, Ilene C. Siegler, Cynthia M. Kuhn, Abanish Singh, Redford B. Williams

**Affiliations:** 1 Department of Psychiatry and Behavioral Sciences, Duke University Medical Center, Durham, North Carolina, United States of America; 2 Center for Human Genetics, Department of Medicine, Duke University Medical Center, Durham, North Carolina, United States of America; 3 Division of Cardiology, Department of Medicine, Duke University Medical Center, Durham, North Carolina, United States of America; 4 Pharmacology and Cancer Biology, Duke University Medical Center, Durham, North Carolina, United States of America; 5 Epidemiological Research and Information Center, Durham VA Medical Center, Durham, North Carolina, United States of America; South Texas Veterans Health Care System and University Health Science Center San Antonio, United States of America

## Abstract

Previously we have shown that a functional nonsynonymous single nucleotide polymorphism (rs6318) of the *5HTR2C* gene located on the X-chromosome is associated with hypothalamic-pituitary-adrenal axis response to a stress recall task, and with endophenotypes associated with cardiovascular disease (CVD). These findings suggest that individuals carrying the rs6318 Ser23 C allele will be at higher risk for CVD compared to Cys23 G allele carriers. The present study examined allelic variation in rs6318 as a predictor of coronary artery disease (CAD) severity and a composite endpoint of all-cause mortality or myocardial infarction (MI) among Caucasian participants consecutively recruited through the cardiac catheterization laboratory at Duke University Hospital (Durham, NC) as part of the CATHGEN biorepository. Study population consisted of 6,126 Caucasian participants (4,036 [65.9%] males and 2,090 [34.1%] females). A total of 1,769 events occurred (1,544 deaths and 225 MIs; median follow-up time =  5.3 years, interquartile range  = 3.3–8.2). Unadjusted Cox time-to-event regression models showed, compared to Cys23 G carriers, males hemizygous for Ser23 C and females homozygous for Ser23C were at increased risk for the composite endpoint of all-cause death or MI: Hazard Ratio (HR)  = 1.47, 95% confidence interval (CI)  = 1.17, 1.84, p  = .0008. Adjusting for age, rs6318 genotype was not related to body mass index, diabetes, hypertension, dyslipidemia, smoking history, number of diseased coronary arteries, or left ventricular ejection fraction in either males or females. After adjustment for these covariates the estimate for the two Ser23 C groups was modestly attenuated, but remained statistically significant: HR  = 1.38, 95% CI = 1.10, 1.73, p = .005. These findings suggest that this functional polymorphism of the *5HTR2C* gene is associated with increased risk for CVD mortality and morbidity, but this association is apparently not explained by the association of rs6318 with traditional risk factors or conventional markers of atherosclerotic disease.

## Introduction

It has been proposed [1] that one way genes influence the development and course of coronary heart disease (CHD) is via moderation of the effects of environmental stressors on brain mechanisms that regulate expression of behavioral and biological endophenotypes that are proximately involved in cardiovascular disease (CVD) pathogenesis and the precipitation of clinical CVD events. The X chromosome gene encoding the serotonin 2C receptor (*5HTR2C*) is an attractive candidate to be playing such a role. This receptor plays a key role in mediation of stress-induced hypothalamic-pituitary-adrenal (HPA) axis activation by stress-induced release of serotonin in the central nervous system (CNS) [2], [3], [4], leading to attempts to identify variants in the *5HTR2C* gene that might moderate the HPA axis response to psychological stress. One such *5HTR2C* variant that has received considerable attention is a coding nonsynonymous single nucleotide polymorphism (SNP) – rs6318; 68G>C – which leads to a substitution of serine for cysteine at codon 23 (Cys23Ser). The frequency of the Ser23 C allele is approximately 13% in unrelated Caucasians [5]. Research indicates that the Ser23 C allele is constitutively more active than the Cys23 G allele [6], with allele-specific analysis of receptor function showing functional differences in the activity of the Ser23 minor allele relative to the Cys23 allele.

Male carriers of the less common Ser23 C allele show a trend toward faster and stronger adrenocorticotropic hormone ACTH responses to the *5HTR2C* agonist m-chlorophenylpiperazine (m-CPP) [7], as well as a different pattern of change in regional cerebral blood flow following m-CPP infusion compared to men carrying the *5HTR2C* Cys23 G allele [8]. In related work [9] we recently found that rs6318 was associated with stress responses to an emotional stress task in the laboratory. Specifically, in a sample of males, Ser23 C allele carriers had two-fold larger increases in both plasma cortisol and subjective ratings of negative emotion during recall of situations that made them angry or sad compared to those carrying the Cys23 G allele. This finding corroborated existing evidence that the *5HTR2C* receptor plays a key role in the activation of the HPA axis by acute stress, and is consistent with prior research [6] showing the Ser23 C allele is constitutively more active than the Cys23 G allele.

Other findings indicate that individuals carrying the *5HTR2C* Ser23 C allele will be more likely than Cys23 G allele carriers to express elevated levels of phenotypes associated with elevated cortisol levels -- central obesity, elevated glucose, insulin and insulin resistance, higher blood pressure and lipids, hypertension, type-2 diabetes, coronary heart disease [10], [11]. Our prior work has also shown that individuals carrying the *5HTR2C* Ser23 C allele are more likely than Cys23 G allele carriers to exhibit metabolic profiles that are related to increased risk levels of CVD (i.e., higher central obesity, poorer glucose metabolism, higher levels of serum lipids). Specifically, in a sample of 243 males and 259 homozygous (C/C vs. G/G) females we found that compared to G allele carriers, C allele carriers who were 30 years of age or older had higher levels of HbA1c and higher hip circumference, total body fat, and trunk fat composition [12]. In addition, irrespective of age, C allele carriers had significantly poorer lipid profiles.

Finally, chronically increased HPA axis function, indexed by elevated hair cortisol levels, has been associated with increased risk of myocardial infarction (MI) in men [13]. Moreover, increased levels of coronary artery calcification have been documented in men and women with larger salivary cortisol responses to mental stress [14]. Based on our model positing that genes influence the development and course of CHD via moderation of stress effects on brain mechanisms that regulate expression of CHD endophenotypes [1] and the evidence reviewed above, we hypothesized that the rs6318 Ser23 C variant would be associated with increased severity of coronary artery disease (CAD) and incidence of CVD mortality and morbidity in a sample of 6,126 Caucasian individuals recruited through the cardiac catheterization laboratory at Duke University Hospital (Durham, NC).

## Methods

### Participants

The collection of CATHeterization GENetics (CATHGEN) subjects has been described elsewhere [15], [16], [17]. Briefly, CATHGEN study participants were recruited through the cardiac catheterization laboratories at Duke University Hospital (Durham, NC, USA), with approval from the Duke Institutional Review Board, and all participants signed informed consent. Although the CATHGEN study is comprised a variety of ethnic groups, the majority of subjects are Caucasian (75.0%). To avoid the confounding effects of population stratification while maximizing our power to detect genetic associations, we chose to analyze the Caucasian participants only. As part of the ongoing follow-up of the Duke Databank for Cardiovascular Diseases (DDCD), CATHGEN participants are surveyed annually for cardiovascular disease and the participant list is compared to the National Death Index. Events were defined as death from any cause and/or MI, and they were followed for up to 11.8 years.

### Measures

**Genotyping.** Genomic DNA was extracted from frozen whole blood samples collected from the participants during cardiac catheterization. SNP was genotyped using Taqman genotyping assay (Life Technologies) and the Type-It Fast Probe PCR kit (Qiagen). For each 5 µl reaction a solution containing 2 µl Type-It Fast Probe PCR Master Mix (Qiagen), 0.05 µl 80x Taqman assay (Life Technologies), and 0.5 µl Q-solution (Qiagen) was added to 3 ng of dried DNA template. The temperature program was 95°C for 5 minutes, then 50 cycles of 95°C for 15 seconds and 60°C for 30 seconds, run on the GeneAmp PCR system 9700 (Life Technologies). Fluorescence was read and analyzed on the ViiA 7 instrument (Life Technologies). PCR reactions were performed in 384 well format with 4 negative controls. SNP genotype call rate was above 95%. Due to the SNP on X chromosome the deviation from Hardy-Weinberg equilibrium was tested only in females, and no such deviation was observed (p = 0.505).

**Risk factors.** Body mass index (BMI), and binary indicators of history of cigarette smoking, dyslipidemia, hypertension, and history of diabetes were all assessed at the time of cardiac catheterization, i.e., at the beginning of the study.

**Disease Severity: Number of diseased vessels and left ventricular ejection fraction.** Severity of CAD was determined as an ordinal variable defined as the number of vessels with significant (≥75%) stenosis as determined by the interventional cardiologist performing the procedure. A higher number of diseased vessels (0–3) indicate greater disease severity. Left Ventricular Ejection Fraction (LVEF) is expressed as the percentage of blood pumped out of the left ventricle on systole. LVEF was measured either during cardiac catheterization, or chart review for an echocardiogram, nuclear study or cardiac MRI done within 1 year on either side of the catheterization.

### Statistical Analyses

We first compared genotypes within each sex on demographic, risk factor, and disease marker variables using linear, logistic or ordinal logistic regression, depending on the measurement characteristics of the response variable. Cox regression models were estimated using a composite of time to all-cause death or verified MI as the response variable. Participants who were lost to follow-up or who had not experienced an event by the last date of follow-up were coded as censored in the analysis. SAS version 9.1 (Cary, NC) and the rms package in R (http://cran-rproject.org) were used for all analyses. Because rs6318 is X-linked, for the purpose of analysis we created a five-level categorical “Sex-Genotype” variable for each combination of sex and genotype (male Ser23 C hemizygotes, male Cys23 G hemizygotes, female Ser23 C homozygotes, female heterozygotes, and female Cys23 G homozygotes). This approach facilitated testing the contrasts of interest, which were: 1) hemizygous Ser23 C males and homozygous Ser23 females versus all Cys23 G carriers; 2) male Ser23 C hemizygotes versus male Cys23 G hemizygotes; 3) female Ser23 C homozygotes vs. female Cys23 G carriers; 4) males versus females. We estimated these contrasts first unadjusted and then adjusted for age, sex, BMI, history of smoking, history of diabetes, history of dyslipidemia, history of hypertension, LVEF, and number of diseased vessels. The proportional hazards assumption was met for all models. A small fraction of cases (0.4%) were missing a measure of BMI and a larger portion (9.9%) were missing a measure of LVEF. In the Cox models, we conducted preliminary analyses comparing multiple imputation with single median imputation for managing these missing cases and found no material difference in the results. We therefore present the results of the models using single median imputation here.

## Results

The clinical characteristics of the CATHGEN study population used in this study are presented for Ser23 C and Cys23 G allele carriers in Table 1, stratified by sex. Variation in rs6318 was not related to any of the demographic, CVD risk, or CVD marker variables. There were a total of 1,544 deaths and 225 non-fatal MIs, totaling to 1,769 clinical events, over a median follow-up time of 5.9 years (interquartile range  =  3.3–8.2). The proportion of individuals experiencing clinical events was highest among male Ser23 C hemizygotes (246/700 = 35.1%) and female Ser23 C homozygotes (23/63 = 36.5%), compared to male Cys23 G hemizygotes (963/3336 = 28.9%), female Cys23 G homozygotes (392/1455 = 26.9%), and female heterozygotes (145/572 = 25.4%).

**Table 1:** Clinical Characteristics by sex and *5HTR2C* rs6318 Ser23 C and Cys23 G allele.

	Males (N = 4,036)			Females (N = 2,090)			
	Ser23 C Hemi-zygotes	Cys23 G Hemi-zygotes	p-value	Ser23 C Homo-zygotes	Ser23C/Cys23G Hetero-zygotes	Cys23G Homo-zygotes	p-value
	N = 700	N = 3,336		N = 63	N = 572	N = 1,455	
Variable	(17.3%)	(82.7%)		(3%)	(27.4%)	(69.6%)	
Age in years, mean (SD)^a^	61.9 (11.2)	61.4 (11.5)	.318	64.5 (11.0)	63.5 (11.8)	63.8 (11.7)	.753
History of smoking, N (%)^b^	408 (58.3%)	1860 (55.8%)	.204	19 (30.2%)	232 (40.6%)	565 (38.3%)	.308
Body Mass Index (kg/m^2^), mean (SD)^ c^	29.4 (6.0)	29.6 (6.1)	.798	30.1 (7.4)	29.8 (8.3)	29.7 (7.9)	.892
History of diabetes, N (%)^b^	202 (28.9%)	847 (25.4%)	.062	12 (19.1%)	143 (25.0%)	374 (25.7%)	.492
History of dyslipidemia, N (%)^b^	476 (68%)	2210 (66.3%)	.409	34 (54.0%)	321 (65.1%)	856 (58.8%)	.446
History of hypertension, N (%)^b^	462 (66.0%)	2229 (66.8%)	.571	42 (66.7%)	386 (67.5%)	971 (66.7%)	.888
Left ventricular ejection fraction (%), mean (SD)^ c^	55.3 (13.2)	55.2 (13.0)	.794	58.9 (9.6)	60.3 (12.0)	60.1 (11.8)	.709
Number of diseased coronary vessels, N (%)^d^							
0	177 (25.3%)	951 (28.5%)	.118	27 (42.9%)	283 (49.5%)	711 (48.9%)	.830
1	169 (24.1%)	785 (23.5%)		20 (31.8%)	142 (24.8%)	340 (23.4%)	
2	135 (19.3%)	645 (19.3%)		8 (12.7%)	70 (12.2%)	224 (15.4%)	
3	219 (31.3%)	955 (28.6%)		8 (12.7%)	77 (13.5%)	180 (12.4%)	

Note. ^a^ p-value derived from unadjusted linear model; ^b^ p-value derived from age-adjusted logistic regression model; ^c^ p-value derived from age-adjusted linear model; ^ d^ p-value was derived from age-adjusted ordinal logistic regression model with number of diseased vessels modeled as an ordinal variable ranging from 0 to 3.

**Figure 1:**
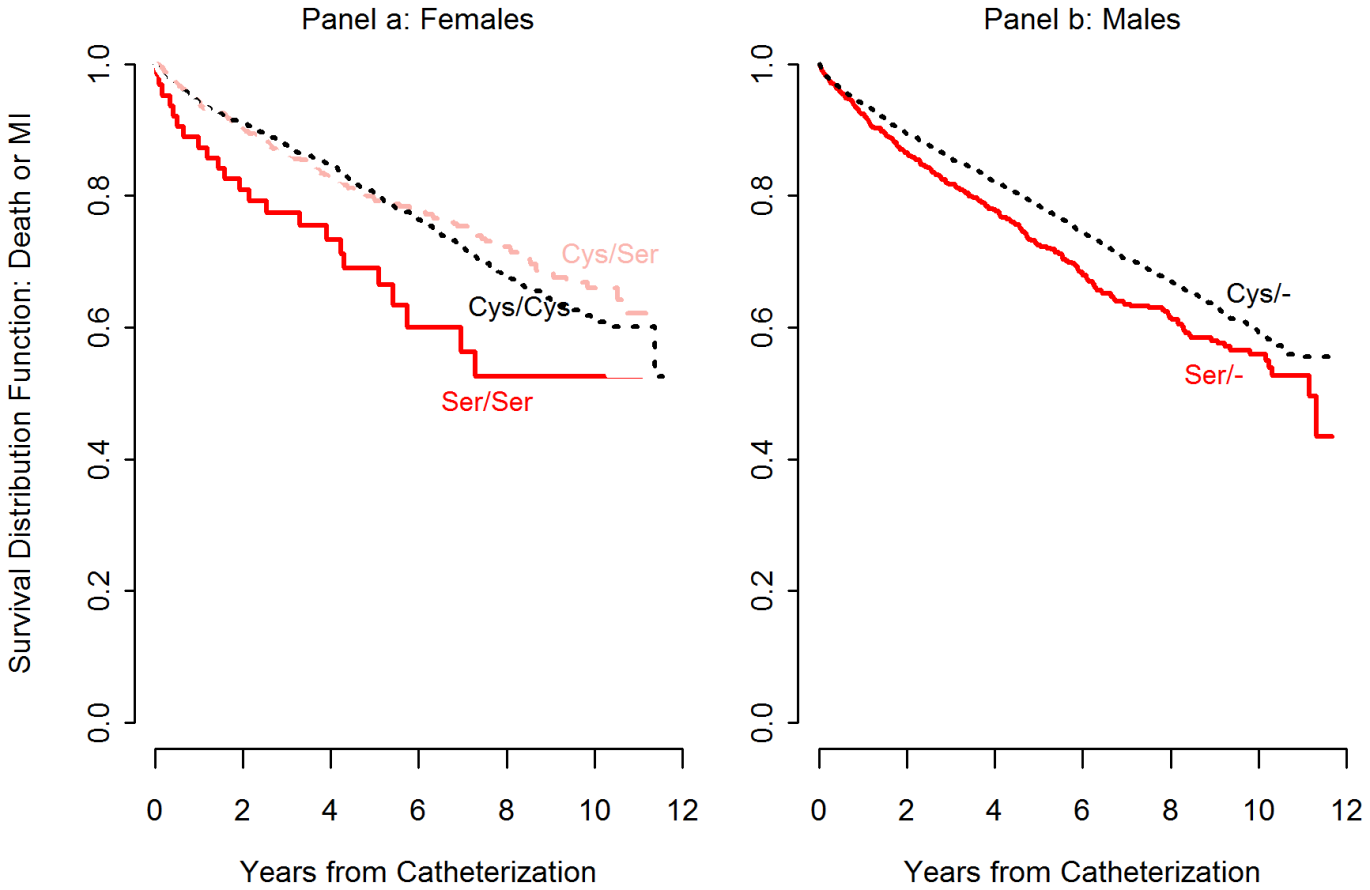
Kaplan-Meier curves illustrating the risk of death or myocardial infarction for each rs6318 genotype in Women (panel a) and Men (panel b). Using pre-specified contrasts in a Cox regression model adjusted for covariates, differences were statistically significant when comparing: male Ser23 C hemizygotes and female Ser23 C homozygotes to all Cys23 G carriers (p  = .005); male Ser23 C hemizygotes to male Cys23 G hemizygotes (p  = .022), and female Ser23 C homozygotes to female Cys23 G carriers (p  = .024).

Unadjusted Kaplan-Meier survival curves for each genotype are presented in Figure 1, separately for each sex (panel a for females, panel b for males). Parallel to the raw event frequency counts, male Ser23 C hemizygotes and female Ser23 C homozygotes showed the most rapid time-to-event rates. These differences in event rates were quantified in the Cox regression models using the entire sample of males and females simultaneously. Table 2 displays the results of each group contrast in the unadjusted and adjusted Cox regression models. In the unadjusted Cox model (columns 1 through 3 in Table 2), compared to all Cys23 G carriers (males hemizygous for Cys23 G, females homozygous for Cys23 G, heterozygous females), hemizygous Ser23 C males and homozygous Ser23C females were at increased risk for the composite clinical endpoint of all-cause death or MI: Hazard Ratio (HR)  = 1.47, 95% confidence interval (CI)  = 1.17, 1.84, p  = .0008. After adjustment for age, BMI, diabetes, hypertension, dyslipidemia, smoking history, number of diseased coronary arteries, and LVEF, the estimate for the two Ser23 C groups was modestly attenuated, but remained statistically significant: HR = 1.38, 95% CI = 1.10, 1.73, p  = .005. Examining contrasts within each sex showed that the increased risk associated with the Ser23 C allele was present for both men and women, but tended to be somewhat larger for women. The hazard ratio from the fully adjusted model for male Ser23 C hemizygotes compared to male Cys23 G hemizygotes was 1.23 (95% CI = 1.02, 1.36, p = 0.02), while the hazard ratio for female Cys23 C homozygotes versus female Cys23 G carriers was 1.62 (95% CI = 1.07, 2.47, p = 0.02). In both cases the unadjusted estimates were modestly attenuated after adjustment for the covariates. No effect for sex *per se* was observed in either adjusted or unadjusted analysis.

**Table 2:** Cox regression models predicting death or non-fatal myocardial infarction (N = 6,126, N events  = 1,769).

	Unadjusted Model			Model Including Adjustment Covariates		
Contrast	Hazard Ratio	95% Confidence Interval	p-value	Hazard Ratio	95% Confidence Interval	p-value
Male Ser23 C hemizygotes and Female Ser23 C homozygotes vs. all Cys23 G carriers	1.47	1.17, 1.84	.0008	1.38	1.10, 1.73	.005
Male Ser23 C hemizygotes vs. Male Cys23 G hemizygotes	1.23	1.07, 1.42	.004	1.18	1.02, 1.36	.022
Female Ser23 C homozygotes vs. Female Cys23 G carriers	1.68	1.11, 2.56	.015	1.62	1.07, 2.47	.024
All Males vs. all Females	1.05	0.98, 1.24	.556	0.92	0.77, 1.09	.312
Age (per 10 year increase)	--	--	--	1.47	1.40, 1.54	<.0001
Body Mass Index (per 7 kg/m^2^ increase)	--	--	--	0.93	0.87, 0.98	<.009
History of Smoking	--	--	--	1.40	1.27, 1.54	<.0001
History of Dyslipidemia	--	--	--	0.75	0.68, 0.83	<.0001
History of Hypertension	--	--	--	1.04	0.93, 1.16	<.485
History of Diabetes	--	--	--	1.49	1.34, 1.66	<.0001
Number of coronary arteries with ≥ 75% stenosis (per one artery increase)	--	--	--	1.14	1.09, 1.19	<.0001
Left Ventricular Ejection Fraction (per 15% increase)	--	--	--	0.74	0.70, 0.78	<.0001

Note: The model including adjustment covariates included all predictors simultaneously.

With the exception of history of hypertension, all of the adjustment covariates were associated with the composite endpoint in the fully adjusted Cox regression model (see Table 2, columns 4 through 6). Older age, history of smoking, history of diabetes, and a higher number of coronary arteries were associated with increased rates of events, while higher BMI, higher left ventricular ejection fraction, and history of dyslipidemia were associated with a lower risk of events.

## Discussion

We have found herein that a functional coding polymorphism in *5HTR2C*, previously associated with enhanced cortisol response to psychosocial stress and intermediate CVD phenotypes, is also associated with a 38% increase in “end” CVD events over a 7-year follow-up period, supporting our hypothesis that mapping of intermediate traits can identify novel CVD genes. However, the status of our hypothesis that the association between Ser23 C and clinical events would be mediated by CVD risk factors or markers of clinical disease is less clear. On one hand, adjustment for these potential mediating variables resulted in modest attenuation of the hazard estimates for Ser23 C, suggesting the possibility of mediation by these variables. On the other hand, however, the lack of direct association between genotype and these potential mediators argues against this interpretation. One possible explanation for this apparent paradox is that the putative mediating variables have an aggregate effect that is not detectable when considering them individually. For example, in males Ser23 C was associated with a slight increase in the frequency of having three vessel CAD and having a history of diabetes, but neither test met the conventional significance criterion of 0.05. In addition, our risk factor variables were indicators of a history of those risk factors rather than direct measures of the risk factors themselves. Since this was a clinical population, many participants may already have been treated for these risk factors, limiting the precision of the history variables as indicators of risk. For example, individuals with histories of dyslipidemia or hypertension may in part be proxies for treatment for these conditions, obscuring the association between these risk factors and the endpoint. Of more certainty is that the persistence of the association between Ser23 C and clinical events even after adjustment suggests the involvement of mechanisms other than those measured in the present study. Enhanced HPA axis response to stress in Ser23 C carriers is one potential candidate mechanism. In our earlier work, we showed that men carrying the Ser23 C allele exhibited larger increases in both HPA axis function as measured by serum cortisol and subjective ratings of anger/sadness in a laboratory setting. In our initial study [9] females were not included due to the low cell counts, with only three women homozygous for the C allele and 16 homozygous for the G allele. Given the relevance of the findings for women in the present study, however, we re-examined the data, now comparing cortisol levels in response to the stress protocol across genotypes. We found that, parallel to the finding in original finding in men, CC women showed larger cortisol response than the GG women (p <.047).

These findings are consistent with the known function of 5HT2C receptors in the hypothalamic paraventricular nucleus (PVN), suggesting that corticotropin-releasing hormone (CRH), when stimulated by serotonin, may be mediating an increase in both HPA axis function and angry and sad moods [9]. In turn, chronic release of endogenous cortisol may be an independent risk factor for cardiovascular clinical events. For example, Pereg et al. [13] recently reported that hair cortisol levels were strongly associated with acute myocardial infarction (OR = OR 17.4, 95% CI = 2.15, 140.45, p  = 0.007) even after adjusting for age, LDL and HDL cholesterol, BMI, smoking status, and previous MI. The authors speculate that the association might be owing to the known effects of cortisol on endothelial function, platelet aggregation, inflammation, or promotion of thrombotic events (see [13], p. 77). Among these potential links between cortisol and clinical events, inflammation may be particularly promising, owing to a recently discovered set of connections from cortisol to unstable atherosclerotic plaque in the coronary arteries. Briefly, unstable plaques are lesions in the vessel endothelium characterized by a lipid-rich core surrounded by a friable cap of connective tissue. As the name implies, vulnerable plaque is easily disrupted by sudden changes in pressure or torsion within the vessel. When the fibrous cap is broken, the lipid content of the plaque is released into the vessel lumen, resulting in thrombus and setting the stage for possible infarction. Several studies have shown that increased cortisol is associated with increased levels of a collagen-degrading protein called matrix metalloproteinase (MMP-9) [18], [19]. MMP-9 in turn has been causally implicated in the development of vulnerable plaque [20]. As noted earlier, rs6318 appears to regulate secretion of cortisol. Thus, a possible, if highly simplified, pathway linking rs6318 to cardiac events may be gene → cortisol → MMP-9 → vulnerable plaque → clinical event. Finally, fatal cardiac arrhythmias also may be a potential mediating mechanism. At present however, we are unaware of a biological link between rs6318 and arrhythmias, nor does our own data set include reliable information on arrhythmias.

Indeed, HPA axis hyperreactivity is regularly cited as a potential mediator of psychosocial risk factors’ health-damaging effects. For example, high levels of hostility have been associated with increased cortisol responses to anger-inducing interpersonal challenge [21], and increased stress in daily life is associated with a higher cortisol awakening response and higher mean day and evening cortisol levels [22]. Investigation of the neurobiological mechanisms by which psychosocial risk factors enhance the activity of the HPA axis indicates that stress-induced release of serotonin in the CNS acts at multiple brain sites to contribute to stress-induced HPA axis activation. These sites include the limbic forebrain including the amygdala, where serotonin stimulates *5HTR2C* receptors on cells in the paraventricular nucleus (PVN), that co-express corticotrophin releasing hormone (CRH), which is released when the *5HTR2C* receptors are stimulated [2], [3], [4]. Thus, the Ser23 C allele’s association with increased cortisol response to emotional stress provides a biologically plausible path that could be responsible for the association we found in this large CVD sample between the Ser23 C allele, possibly by way of CAD severity, but also by way of other mechanisms not captured in our present study.

In addition to its association with enhanced activation of the HPA axis and larger increases in anger and sadness during emotion arousal in the laboratory in our prior study [9], the Ser23 C variant has also been found to be associated with affective disorders [23]. Specifically, in a sample consisting of European populations, there were significantly larger proportions of Ser23 C allele carriers in patients with major depressive and bipolar disorder, as compared to normal controls [23]. Depression has long been linked to dysregulated HPA axis function, and depressed men exhibit higher levels of salivary cortisol across the day compared to healthy men [24]. The present findings, along with studies linking the Ser23 C allele with affective disorders, suggest, therefore, that the rs6318 moderation of 5HT2C receptor-mediated effects on both emotions and the HPA axis may be accounting, at least in part, for the previously reported linkages between depression and dysregulated HPA axis function.

Importantly, increased HPA activation by stress has also been implicated in the impact of stress on the pathogenesis of the metabolic syndrome [25], and elevated cortisol levels have been shown to play a role in mediating the association between depressive symptoms and elevated blood glucose levels [26]. Furthermore, prospective studies provide direct evidence for a causal role in that glucocorticoid excess predicts increased cardiovascular disease (CVD) incidence [27], [28] and elevated cortisol levels have also been found associated with several indices of accelerated aging, including decreased bone mineral density [29], increased incident cognitive impairment [30] and increased frailty[31]. Finally, as noted earlier, we have shown that the Ser23 C allele has been associated with poorer metabolic profiles [12]. Our present analyses, however, showed no association between Ser23 C with BMI, dyslipidemia, or hypertension, three correlates of metabolic syndrome. Again, it may be that because this was a clinical population, participants with dyslipidemia or hypertension may have been adequately treated, thus attenuating any association with these variables. The unique nature of the clinical sample also may be reflected in our observation that history of dyslipidemia and a higher BMI were associated with lower event rates. The unexpected direction of these associations may be a function of adequate treatment for dyslipidemia, or in the case of BMI, excessively low BMI indicating cachexia and advanced disease.

The current findings have potentially important implications for the development of targeted preventive interventions and treatments. It has been shown, for example, that cognitive behavioral stress management training produces reduced anxiety and cortisol levels that are maintained over a 12-month follow-up period in women undergoing treatment for breast cancer [32]. A more direct clinical implication of the current findings is that persons carrying the *5HTR2C* rs6318 Ser23C allele might benefit from treatment with an antagonist of the *5HTR2C* receptor. One such antagonist that has received extensive clinical attention is agomelatine (trade names Valdoxan, Melitor, Thymanax), a melatonergic agonist and 5-HT2C antagonist that has higher efficacy than sertraline in reducing both depressive and anxiety symptoms in depressed patients [33]. Of direct relevance to the current findings, agomelatine has been found to reduce the elevated HPA axis activity produced by psychosocial stress in tree shrews [34]. In healthy older men agomelatine treatment was associated with a lower peak cortisol level reached during the morning hours [35]. It is possible therefore, that treatment with agomelatine could be effective in reducing mortality/MI risk in CHD patients carrying the rs6318 Ser23C allele.

An important limitation of the current study is the fact that this is an association study, as opposed to an experimental design with a direct manipulation. However, it should be noted that our theoretical model and the accumulation of specific findings for the rs6318 Ser23 C allele led to a targeted *a priori* hypothesis that was supported in a large clinical sample. Future work should include attempts at direct replication of the present finding, as well as continued elucidation of the underlying biological mechanisms. In that respect, the currently available evidence for a functional consequence of this SNP comes from reports of differential 5HTR2C function comparing the amino acid substitution of Serine for Cysteine at position 23 [6], [7], [8]. Surveys of available expression quantitative trait loci (eQTL) databases did not, however, identify significant effects of this SNP on gene expression and we found no literature reports of a regulatory role for this SNP on gene expression. Besides effects on gene expression, another possible mechanism that could account for the effects of the rs6318 C allele on the function of the 5HTR2C receptor that increase cortisol responses to stress (and thereby affect clinical outcomes in CHD patients) is that substitution of serine for a cysteine residue in 5HTR2C protein among C allele carriers could result in disruption of disulfide bridges that play a critical role in the folding of a protein into its native conformation [36]. More research, beyond the scope of this report, will be required to determine whether protein structure and folding varies as a function of rs6318 genotype. Finally, the present findings were limited to Caucasian participants and may not generalize to populations of other ethnicities.

In conclusion, we found that a previously documented functional SNP, rs6318, on the *5HTR2C* gene that has been previously associated with increased cortisol response to stress, depression, and metabolic traits linked to CVD, was associated with increased incidence of death or MI, independent of several traditional risk factors and two conventional markers of CVD severity in a large sample of Caucasian patients. If replicated in further research, these findings suggest that behavioral and pharmacologic interventions to reduce HPA axis hyperreactivity in rs6318 Ser23C allele carriers deserve attention as a means of improving CVD-related prognosis in patients, and ultimately in primary prevention.
